# Prospective investigation of positive psychotic symptoms, dissociative symptoms, and metacognitive dysfunctions in a non-clinical population

**DOI:** 10.1038/s41598-025-19547-w

**Published:** 2025-10-13

**Authors:** Bryan Ho-wang Yu, Anson Kai Chun Chau, Chui-De Chiu, Suzanne Ho-wai So

**Affiliations:** 1 Department of Psychology, The Chinese University of Hong Kong, Hong Kong SAR, China; 2 Department of Psychiatry, The University of Hong Kong, Hong Kong SAR, China

**Keywords:** Psychotic experiences, Dissociation, Psychosis, Metacognition, Structural equation modelling, Cross-lagged panel model, Psychology, Risk factors

## Abstract

The frequent co-occurrence of positive psychotic and dissociative symptoms in both clinical and non-clinical populations underscores the importance of understanding their interplay for early intervention. However, their longitudinal dynamics and shared risk factors in non-clinical individuals are poorly understood. This prospective study investigated the reciprocal relationship between positive psychotic and dissociative symptoms and the role of metacognitive dysfunction in 2,360 adults without psychiatric diagnoses (age 18–65). Participants completed online questionnaires at baseline and at six months, reporting levels of positive psychotic and dissociative symptoms, maladaptive metacognitive beliefs and metacognitive functioning. Longitudinal structural equation modelling, controlling for autoregressive effects, assessed the variables’ cross-lagged associations. Results showed that baseline positive psychotic symptoms predicted dissociative symptoms at six months, and vice versa. Maladaptive metacognitive beliefs (lower cognitive self-consciousness) and poorer metacognitive functioning (self-reflectivity) at baseline predicted increased dissociative symptoms, but not positive psychotic symptoms, at six months. Higher baseline dissociative symptoms reciprocally predicted poorer metacognitive functioning at six months. Our findings support the mutual influence between positive psychotic and dissociative symptoms, and the specific association of metacognitive factors with dissociative symptoms, independent of co-occurring positive psychotic symptoms. Future research should extend these findings with longer follow-up periods and in clinically diverse groups.

**Supplementary Information:**

The online version contains supplementary material available at 10.1038/s41598-025-19547-w.

## Introduction

Psychosis and dissociation are transdiagnostic, debilitating phenomena. Psychosis comprises positive (e.g., delusions, hallucinations), negative (e.g., avolition, diminished affect), and disorganisation symptoms. Dissociation denotes disruption and/or discontinuity of the normal integration of consciousness, perception, memory, and identity (e.g., depersonalisation, amnesia). Although theoretically and diagnostically distinct^1^, compelling evidence demonstrates robust association between positive psychotic and dissociative symptoms across clinical and non-clinical populations^2,3^, with notable co‑occurrence rates in patient cohorts^4–6^ and in the general population^7^. Co-occurrence of these symptoms is associated with poorer clinical outcomes than either symptom cluster alone^7–9^.

This empirical interrelationship warrants investigation of their dynamic interplay^10^. Experience sampling and longitudinal studies indicate that dissociation predicts later positive psychotic symptoms in patients with psychotic disorders^11,12^, plausibly via impaired cognitive inhibition^13^ or reality-testing^14^ that renders intrusions and anomalous experiences salient and alien^15,16^. Conversely, positive psychotic symptoms are hypothesised to elicit dissociative responses by precipitating threat-related arousal or perceptual incoherence^10,17,18^especially during an acute psychotic episode^19^; however, this direction is less studied.

Recent studies of the bidirectional relationship between positive psychotic and dissociative symptoms, using different methods and samples within the general population, yielded inconsistent findings. A directed network analysis suggested that dissociation likely predicts paranoia^20^, but the reverse direction was less conclusive. A prospective study^21^ reported a dissociative-to-positive psychotic link in a small Chinese-speaking sample but not their English-speaking sample; contrarily, a multi-wave adolescence cohort study^22^ reported a cross-lagged effect of psychosis on dissociation. These studies, however, are limited by inconsistent within-study assessment methods (e.g., different measures and follow-up intervals across samples^21^, self-reports for certain symptoms versus caregiver ratings for other symptoms^22^) and use of a dissociation measure assessing mostly non-pathological absorption only^22,23^. These mixed findings underscore the need for a carefully designed investigation into the bidirectional symptom relationship among diagnosis-free individuals.

Dysfunction in metacognition, the capacity to reflect on one’s own and others’ thinking^24^, offers a theoretical framework for the investigation of the psychopathology of psychosis and dissociation. Two constructs are relevant: maladaptive metacognitive beliefs^25^ (e.g., beliefs about uncontrollability or the need to control thoughts) and metacognitive functioning^26^ (abilities including selfreflectivity, understanding others’ minds, and maintaining ‘critical distance’ between subjective mental representations and the reality). Maladaptive metacognitive beliefs bias how we monitor and regulate our cognitions and safety behaviours^27^, potentially amplifying awareness and appraisal of anomalous experiences (e.g., heightened sense of ‘cognitive selfconsciousness’ or a ‘need to control thoughts’), fostering persistence of psychotic and dissociative phenomena^17,28–30^. Deficits in metacognitive functioning, on the other hand, impair our ability to integrate discrete experiences into a coherent representation of the self and others^31,32^. This may increase vulnerability to positive psychotic symptoms by distorting interpretations of inner mental events and external events for a proper explanation of the reality^33,34^. While individuals with dissociative disorders often present fragmented self-representations^35^, this could stem from deficits in metacognitive monitoring or integration^36^, affecting processing of self-referenced materials^37–39^ and emotion recognition (i.e., alexithymia)^40^.

While metacognitive dysfunction is implicated in positive psychotic and dissociative symptoms, there remains knowledge gap. First, most studies examine metacognitive dysfunction in these conditions separately, leaving it unclear which specific metacognitive dysfunction can differentiate symptom clusters or predict their co-occurrence, hindering more precise targets for assessment and interventions^41,42^. For example, biased ‘cognitive confidence’ (a maladaptive metacognitive belief^25^) may be a differentiating factor because psychotic disorders involve impaired reality testing yet *inflated confidence* despite erroneous responses^43^, whereas dissociation is linked to *decreased confidence* in cognitive performance^44^. Also, diminished self-reflectivity (a metacognitive function) may contribute to both symptom types due to their common presentation of self-disturbances^38^, but deficits in “understanding others’ minds” and ‘critical distance’ may better predict positive psychotic symptoms, as dissociative disorders usually involve less impaired social cognition^45,46^. Second, although typically viewed as an antecedent risk factor, metacognition may not be strictly trait-like; it can improve or deteriorate following therapeutic interventions^24^ or mood disturbances^47^. Hence, positive psychotic and dissociative symptoms may exhibit a reciprocal relationship with metacognitive dysfunction as well.

Currently, most studies on the interplay between positive psychotic and dissociative symptoms and metacognitive dysfunctions were either cross-sectional^29,33^ or focused on clinical samples only^28,34^; longitudinal studies in non‑clinical adults remain sparse. Studying non‑clinical populations offers an advantage: it allows examination of how symptoms and risk factors emerge and interact prior to disorder onset, improving understanding of early mechanisms and potential targets for prevention. To address these gaps, this study used repeated measures (two timepoints, 6 months apart) and longitudinal structural equation modelling (SEM) to delineate the temporal interplay between positive psychotic and dissociative symptoms, and metacognitive dysfunctions in a large, non-clinical adult sample. This design permits robust testing of cross-lagged relationships between latent variables (correcting for measurement error) through a model comparison approach. The study hypotheses were:

1. Positive psychotic symptoms at baseline will predict an increase in dissociative symptoms at follow-up, and vice versa.
2. Metacognitive beliefs ‘need to control thoughts’, ‘uncontrollability and danger of thoughts’, and ‘cognitive self-consciousness’ at baseline will predict increases in positive psychotic and dissociative symptoms at follow-up, whereas ‘cognitive confidence’ at baseline will predict an increase in dissociative symptoms only at follow-up.
3. Metacognitive functioning ‘self-reflectivity’ at baseline will predict increases in positive psychotic and dissociative symptoms at follow-up, whereas “understanding others’ mind” and ‘critical distance’ at baseline will predict an increase in positive psychotic symptoms only at follow-up.

## Method

The study was pre-registered with OSF Registries (https://osf.io/pvuws). Ethics approval was obtained from the Survey and Behavioural Research Ethics Committee of The Chinese University of Hong Kong (Ref. No.: SBRE-22-0215). The study adhered to ethical standards set by national and institutional committees and the Declaration of Helsinki. All participants gave written informed consent.

## Participants and procedures

Non-clinical adults aged 18–65 who resided in Hong Kong and could read Traditional Chinese were recruited from the general population, through mass emailing in local universities, snowball sampling, Facebook, and distribution of pamphlets around local communities. Participants with current or previous psychiatric diagnosis (by self-report) were excluded. At baseline and 6-month follow-up, participants completed an online survey assessing demographics, positive psychotic symptoms, dissociative symptoms, and metacognitive variables. Target sample size was estimated using the R Package ‘semPower’^48^. Assuming medium autoregressive effects and small cross-lagged effects, with α = 0.05 and a power of 0.80, at least 1,543 participants would be needed for cross-lagged panel model SEM analysis.

## Measures

### Positive psychotic symptoms

Positive psychotic symptoms were indexed by the three subscores of the Community Assessment of Psychic Experiences-Positive Scale (CAPE-P15)^49^. The CAPE-P15 is a brief version of the original CAPE^50, ^assessing three dimensions of positive psychotic symptoms: ‘persecutory ideations’, ‘bizarre experiences’, and ‘perceptual abnormalities’. Level of frequency of each item is rated using a 4-point scale from 1 (‘never’) to ‘4 (‘nearly always’). Good internal consistencies were obtained for the total score (α = 0.834) and subscores (α = 0.704–0.758) in the current sample.

### Dissociative symptoms

Dissociative symptoms were indexed by the five subscores of the Dissociative Experience Measures Oxford (DEMO)^51^. The 30-item DEMO measures five dimensions of dissociative symptoms: ‘unreality’, ‘numbness/disconnect’, ‘memory blanks’, ‘zone-out’, and ‘vivid internal world’. Each item is rated on a 5-point scale from 1 (‘not at all’) to 5 (‘most of the time’). Excellent internal consistencies were achieved for the total score (α = 0.942) and subscores (α = 0.805–0.894) in the current sample.

### Maladaptive metacognitive beliefs

The Metacognitions Questionnaire–Short Form (MCQ-30)^25^ measures five domains of maladaptive metacognitive beliefs: positive beliefs about worry (PB), uncontrollability and danger of thoughts (UD), cognitive self-consciousness (CSC), need to control thoughts (NC), and cognitive confidence (CC) on a 4-point scale from 1 (‘do not agree’) to 4 (‘strongly agree’). The current sample reported good internal consistencies for the total score (α = 0.903) and subscores (α = 0.739–0.876). Each domain of maladaptive beliefs was indexed by the corresponding subscale items.

#### Metacognitive functioning

The 18-item Metacognition Self-Assessment Scale (MSAS)^52^ comprises four subscales measuring self-reflectivity (SELF), critical distance (CrDis), understanding other’s mind (UOM), and mastery (M) on a 5-point scale from 1 (‘never’) to 5 (‘almost always’). Poorer functioning is indicated by lower scores. The current sample yielded excellent internal consistencies for the total score (α = 0.938) and subscores (α = 0.841–0.889). Each domain of metacognitive functioning was indexed by the corresponding subscale items.

## Statistical analysis

Only participants who completed the survey at both time points were included in the analyses. Response validity was verified following Curran et al.^53^ Responses were excluded if they (1) were duplicates; (2) failed three out of five attention checks; and (3) had a completion time below the product of the number of items×2s^54^.

Data analyses were conducted using IBM SPSS 28 and the ‘lavaan’ package^55^ on R. Descriptive statistics of demographics and correlations between study variables were followed by Confirmatory Factor Analyses (CFA) to assess the latent constructs and measurement invariance across time. As longitudinal metric invariance was established for CAPE-P15 and DEMO but not for MCQ-30 and MSAS (see Supplementary information), factor loadings of positive psychotic and dissociative symptoms were fixed to be equal across waves.

The prospective relationships between latent variables were examined using longitudinal SEM and a model comparison approach. First, all variables at follow-up were regressed on their values at baseline (i.e. autoregressive paths; models A0/B0/C0). The next models included additional cross-lagged paths in a stepwise manner. Hypothesis 1 was tested using a cross-lagged panel model between positive psychotic and dissociative symptoms (models A1-3). For Hypotheses 2–3, examination of successive models with autoregressive paths plus cross-lagged paths (i) between symptoms, (ii) from metacognitive dimensions to symptoms, and (iii) from symptoms to metacognitive dimensions were repeated for metacognitive beliefs (models B1-3) and metacognitive functioning (models C1-3) respectively. The final models (B4, C4) incorporated significant path(s) from the above models.

Parameters were estimated using robust maximum likelihood (MLR). Model fit was evaluated using the chi-square statistic, Comparative Fit Index (CFI), Root Mean Square Error of Approximation (RMSEA), and Standardised Root Mean Squared Residual (SRMR). A CFI value > 0.90 is acceptable and > 0.95 is excellent, and RMSEA and SRMR values < 0.10 are acceptable and < 0.05 are excellent^56^. Competing nested models were compared using the Satorra-Bentler scaled chi-square difference test^57^ and Akaike’s Information Criterion (AIC) with a lower score indicating a better fit.

## Results

### Demographic characteristics

**Fig. 1:**
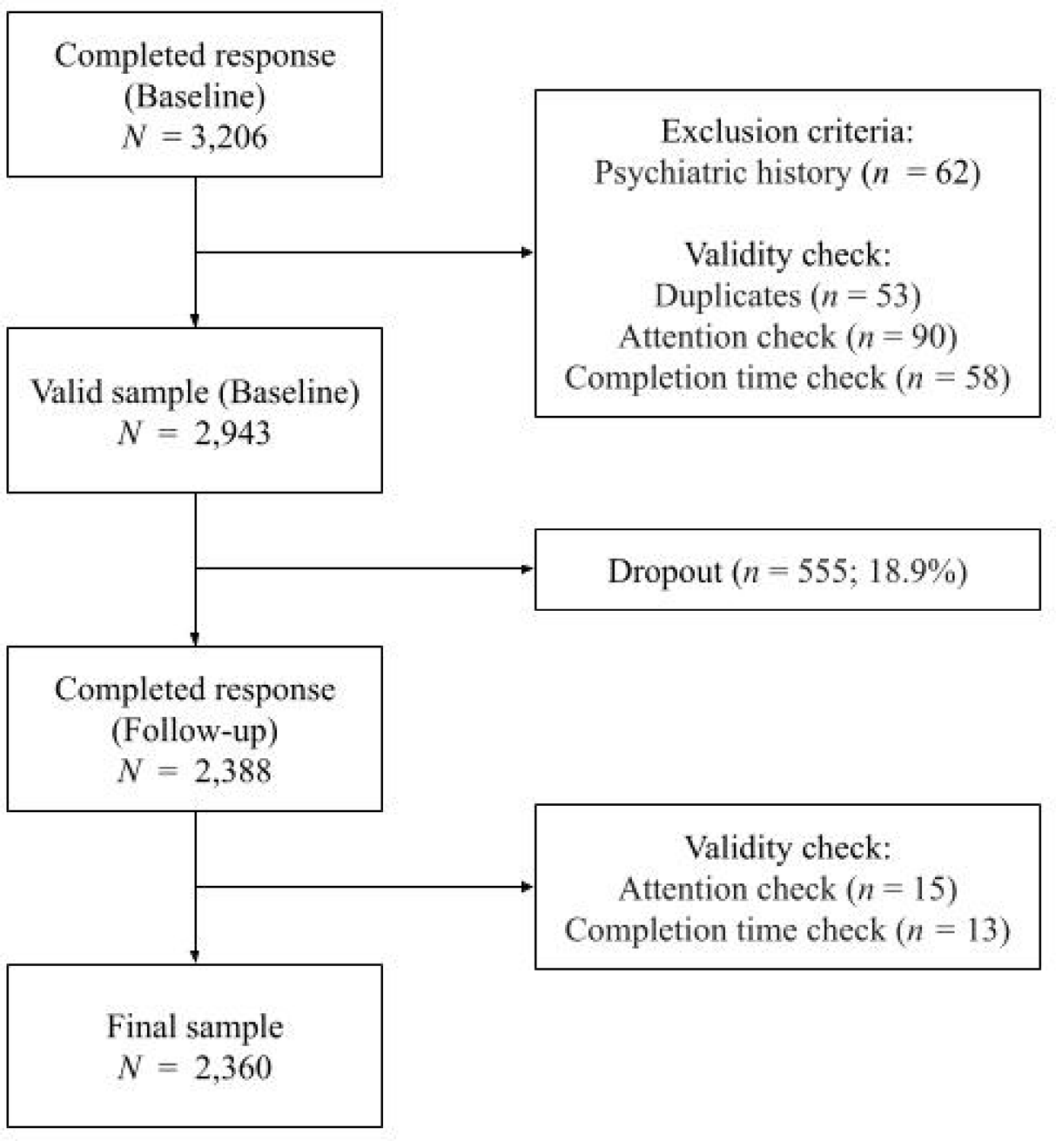
Consort diagram of the sampling procedure.

The final valid sample consisted of 2,360 participants who completed the survey at both timepoints (see Fig. 1 for details). The sample had a mean age of 34.92 (SD = 12.59); the majority was female (73.3%), currently employed (63.8%), and received tertiary education (76.1%). Descriptive statistics for positive psychotic symptoms, dissociative symptoms, and metacognition at each timepoint are shown in Table 1.

**Table 1:** Key variables at each timepoint (N = 2,360).

	Mean (SD)		Follow-up vs. baseline (Paired sample t-test)
	Baseline	Follow-up	
CAPE-P15			
Total	21.289 (4.707)	21.038 (4.605)	*t =* 3.258, *p =* 0.001
Persecutory ideation	8.362 (2.198)	8.182 (2.104)	*t =* 4.760, *p <* 0.001
Bizarre experiences	9.664 (2.708)	9.576 (2.643)	*t =* 1.865, *p =* 0.062
Perceptual abnormalities	3.263 (0.771)	3.279 (0.777)	*t =* -0.971, *p =* 0.332
DEMO			
Total	50.070 (15.472)	50.114 (15.873)	*t =* -0.180, *p =* 0.857
Unreality	8.652 (3.656)	8.560 (3.669)	*t =* 1.454, *p =* 0.146
Numbness/disconnectedness	11.088 (4.701)	11.100 (4.448)	*t =* -0.162, *p =* 0.872
Memory blanks	8.269 (3.204)	8.595 (3.110)	*t =* -5.649, *p <* 0.001
Zone-out	10.505 (4.198)	10.817 (4.044)	*t =* -4.441, *p <* 0.001
Vivid internal world	11.557 (30.809)	11.042 (3.267)	*t =* 7.455, *p <* 0.001
MCQ-30			
Total	57.269 (12.394)	60.133 (12.621)	*t =* -13.461, *p <* 0.001
Positive beliefs about worry	10.603 (3.538)	11.201 (3.680)	*t =* -8.928, *p <* 0.001
Uncontrollability and danger	11.268 (3.572)	11.843 (3.772)	*t =* -9.142, *p <* 0.001
Cognitive self-consciousness	13.467 (3.611)	14.231 (3.462)	*t =* -7.611, *p <* 0.001
Need to control thoughts	10.672 (3.140)	11.150 (3.275)	*t =* -11.257, *p <* 0.001
Cognitive confidence	11.259 (3.888)	11.708 (4.030)	*t =* -7.819, *p <* 0.001
MSAS			
Total	63.368 (11.601)	67.251 (11.245)	*t =* -18.047, *p <* 0.001
Self-reflectivity	17.796 (3.962)	19.200 (3.717)	*t =* -17.996, *p <* 0.001
Understanding other’s mind	9.747 (2.385)	10.354 (2.396)	*t =* -12.622, *p <* 0.001
Critical distance	18.528 (3.701)	19.398 (3.498)	*t =* -12.118, *p <* 0.001
Mastery	17.297 (3.623)	18.298 (3.650)	*t =* -13.561, *p <* 0.001

CAPE-P15 = Community Assessment of Psychic Experiences-Positive Scale; DEMO = Dissociative Experience Measures Oxford; MCQ-30 = Metacognitions Questionnaire-Short Form; MSAS = Metacognitive Self-Assessment Scale.

### Relationship between positive psychotic and dissociative symptoms over 6 months

**Table 2:** Fit statistics and model comparisons for nested models.

Model		χ^2^ (df)	CFI	RMSEA	SRMR	AIC	Model comparison	Chi-square difference test (Δχ^2^ (df))
Cross-lagged symptoms								
A0:	Autoregressive paths	546.389 (98), *p* < 0.001	0.969	0.044	0.037	161235.705	-	-
A1:	A0 + DEMO → CAPE-P15	519.918 (97), *p* < 0.001	0.971	0.043	0.036	161193.764	A1 vs. A0	21.945 (1), *p* < 0.001
A2:	A0 + CAPE-P15 → DEMO	527.442 (97), *p* < 0.001	0.971	0.043	0.036	161207.575	A2 vs. A0	18.449 (1), *p* < 0.001
A3:	A0 + DEMO ←→ CAPE-P15	514.195 (96), *p* < 0.001	0.972	0.043	0.035	161186.584	A3 vs. A0	29,0.439 (2), *p* < 0.001
Models added maladaptive metacognitive beliefs								
B0:	Autoregressive paths	11349.582 (2693), *p* < 0.001	0.898	0.037	0.071	437586.235	-	-
B1:	B0 + DEMO ←→ CAPE-P15	11313.120 (2691), *p* < 0.001	0.898	0.037	0.071	437542.520	B1 vs. B0	26.408 (2), *p* < 0.001
B2:	B0 + MCQ-30 subscales → symptoms	11313.387 (2683), *p* < 0.001	0.898	0.037	0.070	437565.452	B2 vs. B0	36.048 (10), *p* < 0.001
B3:	B0 + symptoms → MCQ-30 subscales	11318.781 (2683), *p* < 0.001	0.898	0.037	0.070	437565.789	B3 vs. B0	31.807 (10), *p* < 0.001
B4:	B0 + MCQ-30 subscales ←→ symptoms	11304.826 (2690), *p* < 0.001	0.898	0.037	0.071	437535.541	B4 vs. B0	36.798 (3), *p* < 0.001
Models added metacognitive functioning								
C0:	Autoregressive paths	5920.882 (1218), *p* < 0.001	0.929	0.040	0.060	333552.089	-	-
C1:	C0 + DEMO ←→ CAPE-P15	5889.552 (1216), *p* < 0.001	0.930	0.040	0.060	333512.620	C1 vs. C0	23.715 (2), *p* < 0.001
C2:	C0 + MSAS subscales → symptoms	5899.166 (1210), *p* < 0.001	0.930	0.041	0.057	333544.973	C2 vs. C0	20.653 (8), *p* = 0.008
C3:	C0 + symptoms → MSAS subscales	5822.672 (1210), *p* < 0.001	0.931	0.040	0.045	333447.347	C3 vs. C0	93.336 (8), *p* < 0.001
C4:	C0 + MSAS subscales ←→ symptoms	5780.735 (1211), *p* < 0.001	0.931	0.040	0.044	333396.388	C4 vs. C0	132.180 (7), *p* < 0.001
C5:	C4 with non-significant paths removed	5782.631 (1212), *p* < 0.001	0.931	0.040	0.044	333395.571	C5 vs. C4	1.228 (1), *p* = 0.268

CAPE-P15 = Community Assessment of Psychic Experiences-Positive Scale; DEMO = Dissociative Experience Measures Oxford; MCQ-30 = Metacognitions Questionnaire-Short Form; MSAS = Metacognitive Self-Assessment Scale. Arrows represent the predictive direction of the cross-lagged path(s) between variables.

**Fig. 2:**
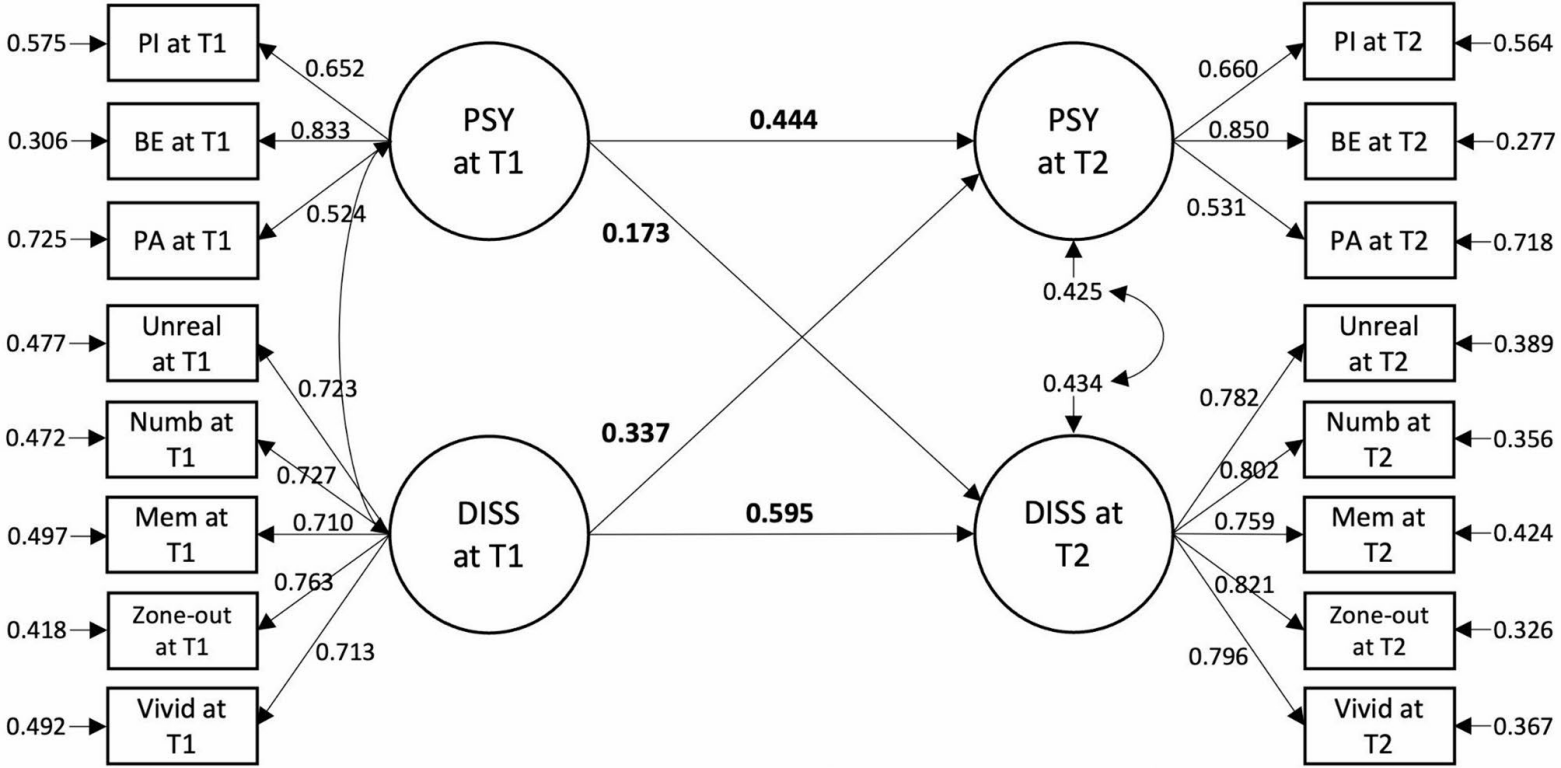
Final structural equation model of positive psychotic and dissociative symptoms across time points. The diagram represents model A3 (see Table 2). Values given represent standardised coefficients.* T1 * baseline;* T2*  6 months. *PSY * Positive psychotic symptoms; *PI* Persecutory ideation;* BE* Bizarre experiences;* PA* Perceptual abnormalities;* DISS* Dissociative symptoms;* Unreal* Unreality;* Numb* Numbness/disconnectedness;* Mem* Memory blanks;* Vivid* Vivid internal world. Only significant paths are shown (*ps* < 0.050).

Model fit indices and model comparisons of the longitudinal SEM models are listed in Table 2. The model incorporating full autoregressive and cross-lagged paths (Model A3) between positive psychotic and dissociative symptoms across time yielded the best model fit (see path coefficients in Fig. 2). Controlling for autoregressive effects (*ps* < 0.001), dissociative symptoms at baseline significantly predicted an increase in positive psychotic symptoms at follow-up (β = 0.337, *p* < 0.001), and vice versa (β = 0.173 *p* = 0.017). Comparison between cross-lagged paths suggested no significant differences in their standardised regression weights (∆β = 0.164, *p* = 0.291).

### Relationship between symptoms and maladaptive metacognitive beliefs over 6 months

**Fig. 3:**
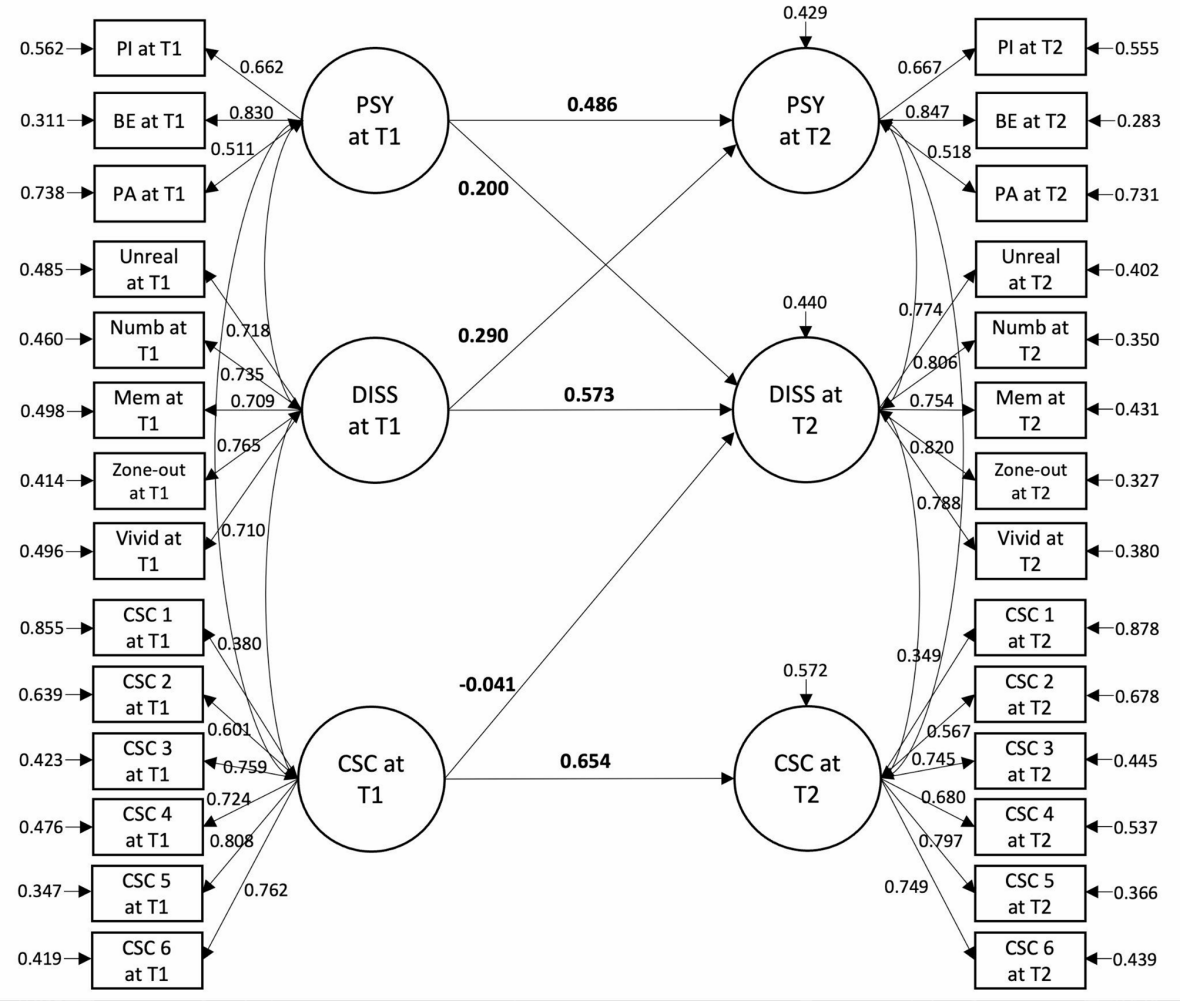
Final structural equation model of positive psychotic symptoms, dissociative symptoms, and maladaptive metacognitive beliefs across time points. The diagram represents model B4 (see Table 2). Values given represent standardised coefficients. Autoregressive paths of other dimensions of the Metacognition Questionnaires-Short Form (positive beliefs about worry, uncontrollability and danger of thoughts, need to control thoughts, cognitive confidence) that did not have cross-lagged associations with symptoms are not shown in this figure for cleaner presentation.* T1 * baseline,* T2*  6 months.  *PSY * Positive psychotic symptoms;* PI* Persecutory ideation;* BE* Bizarre experiences;* PA* Perceptual abnormalities;* DISS* Dissociative symptoms;* Unreal* Unreality;* Numb* Numbness/disconnectedness;* Mem* Memory blanks;* Vivid* Vivid internal world;* CSC* Cognitive self-consciousness. Only significant paths are presented (*ps* < 0.050).

The full SEM model (Model B4) yielded an acceptable model fit (Fig. 3; Table 2). The cross-lagged relationship between positive psychotic and dissociative symptoms across time remained significant (*ps* < 0.050). The CSC subscale of MCQ-30 at baseline negatively predicted dissociative symptoms at follow-up (β = -0.041, *p =* 0.003). None of the metacognitive beliefs at baseline predicted positive psychotic symptoms at follow-up (*ps* > 0.050). Symptoms at baseline did not predict maladaptive metacognitive beliefs at follow-up.

### Relationship between symptoms and metacognitive functioning over 6 months

**Fig. 4:**
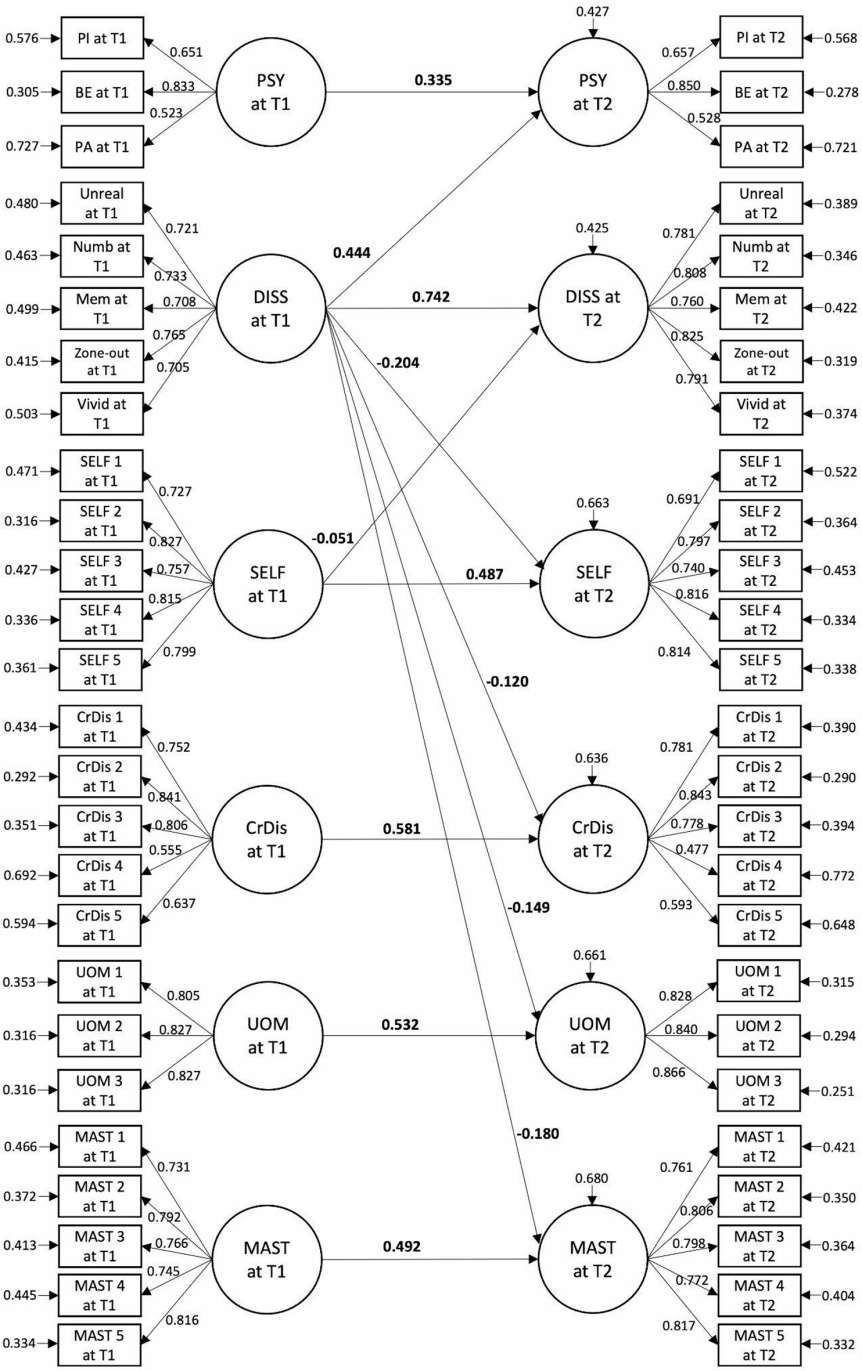
Final structural equation model of positive psychotic symptoms, dissociative symptoms, and metacognitive functioning across time points. The diagram represents model C5 (see Table 2). Values given represent standardised coefficients. Covariance paths among latent variables are not shown in this figure for cleaner presentation.* T1 * baseline,* T2 * 6 months.* PSY* Positive psychotic symptoms;* PI * Persecutory ideation;* BE* Bizarre experiences;* PA * Perceptual abnormalities;* DISS* Dissociative symptoms;* Unreal* Unreality;* Numb* Numbness/disconnectedness;* Mem* Memory blanks;* Vivid* Vivid internal world;* SELF* Self-reflectivity;* CrDis* Critical distance;* UOM* Understanding other’s mind;* MAST* Mastery. Only significant paths are presented (*ps* < 0.050).

The initial full SEM model (Model C4) showed a good model fit (Table 2), but some paths became non-significant and were thus removed. The final model (Model C5) also yielded a good model fit (Fig. 4; Table 2). Baseline dissociative symptoms significantly predicted positive psychotic symptoms at follow-up (β = 0.444, *p <* 0.001), but the cross-lagged path from positive psychotic symptoms to dissociative symptoms was not significant. None of the metacognitive functioning dimensions at baseline predicted positive psychotic symptoms at follow-up (*ps* > 0.050) when the path from dissociative symptoms to positive psychotic symptoms was added. Only MSAS self-reflectivity at baseline significantly and negatively predicted dissociative symptoms at follow-up (β = -0.051, *p =* 0.001). Reciprocally, with the autoregressive effects controlled, dissociative symptoms at baseline negatively predicted all dimensions of metacognitive functioning at follow-up (βs = -0.151 to -0.251, *ps* < 0.001). Comparison between paths suggested that the standardised regression weight of the path from dissociative symptoms at baseline to self-reflectivity at follow-up was significantly stronger than the opposite direction (∆β = 0.153, *p* < 0.001).

Post-hoc analyses were run after controlling for age, gender, and levels of depression at both timepoints. All cross-lagged effects in the final models (A3, B4, C5) remained significant (*ps* < 0.050).

## Discussion

To clarify the interplay between positive psychotic and dissociative symptoms in non-clinical individuals, this study employed repeated measures and longitudinal SEM with a model comparison approach. We tested cross‑lagged symptom relationships and the prospective effects of maladaptive metacognitive beliefs and metacognitive functioning over six months.

Our study provides the first prospective evidence of a reciprocal relationship between positive psychotic and dissociative symptoms, with comparable cross‑lagged effects, in diagnosis-free individuals. Strengths includes a large sample and comprehensive assessment of dissociation^51^. While baseline dissociative symptoms predicted changes in positive psychotic symptoms prospectively, aligning with previous clinical research^11,12^, baseline positive psychotic symptoms also predicted subsequent changes in dissociative symptoms – indicating that presence of one symptom cluster may increase risk of the other. Critically, however, the psychosis-to-dissociation path became non-significant upon inclusion of metacognitive functioning, implying this link as less robust or accounted for by shared variance with other factors.

The reciprocal effect should be interpreted with caution given that changes in symptoms were observational rather than experimentally manipulated. Moreover, empirical tests of mediating mechanisms between positive psychotic and dissociative symptoms remain scarce; future research should prioritise experimental and process‑focused studies to identify pathways (e.g., cognitive, affective) that underlie these constructs. It is also important to recognise that positive psychotic symptoms (delusions, hallucinations) and dissociative symptoms (detachment and compartmentalisation^58^ are heterogenous, and that individual symptoms may relate to each other in varied ways^59^. Clarifying these nuanced pathways (e.g., via subgroup analyses or symptom‑level network models) may be beneficial to delineate distinct symptom‑specific mechanisms.

For the first time, metacognitive dysfunction was shown to be specifically associated with dissociative symptoms after accounting for autoregression and co‑occurring positive psychotic symptoms. The cognitive self-consciousness (CSC) domain of maladaptive metacognitive beliefs negatively predicted changes in dissociative symptoms: lower self‑focused attention on cognitive processes was linked to subsequent increases in dissociative symptoms. Although the effect size was small, this contrasts with previous cross-sectional work that reported positive links between heightened self-consciousness and dissociation^30,60^. Indeed, Spearman’s rho correlations in our data were positive crosssectionally at each time point. Future longitudinal studies should investigate this apparent divergence between cross‑sectional and prospective associations and test mechanisms that could account for an inverse CSC-dissociation trajectory.

Another novel finding was that deficits in metacognitive functioning, particularly self‑reflectivity, and dissociative symptoms exhibited reciprocal prediction over six months, suggesting bidirectional influences rather than a unidirectional pathway. This suggests that dissociative symptoms arise from and reciprocally disrupt the ability to understand one’s own mind. Self-reflectivity involves identifying and relating the various components of our mental state (e.g., thoughts, emotions, memories, motivations), and continuously integrating them into coherent and continuous aspects of the self^31^. Deficits in self-reflectivity may therefore lead to reduced access to self-referenced representations, a crucial mechanism contributing to dissociation proneness^37^, as it prevents compartmentalised information, like early traumatic events, from assimilating into existing autobiographical experiences. Conversely, experiencing dissociative symptoms may diminish one’s sense of individuality and compromise self-reflective abilities as well. For instance, dissociation is linked to biases in self-other source monitoring^38^, which may indicate an underlying deficit in metacognitive monitoring and integration of components of self-generated representations.

Although prior research implicates that metacognitive dysfunction is central to schizophrenia^46,61^ and contributes to prolonged positive psychotic symptoms^28,34^, neither maladaptive metacognitive beliefs nor deficits in metacognitive functioning were predictive of positive psychotic symptoms in our model. Several explanations are plausible. Firstly, our non-clinical sample had lower positive psychotic symptoms and preserved metacognitive performance than clinical samples^62,63^, hence associations evident in clinical samples may be absent here^64^. Secondly, maladaptive metacognitive beliefs may relate selectively to paranoid ideations but not hallucinations^65,66^; aggregating the symptoms into a single latent variable in this study could have prevented such symptom-specific links from being detected. Thirdly, both metacognitive variables predicted dissociative symptoms, which in turn predicted positive psychotic symptoms, raising the possibility that dissociative symptoms mediate effects of metacognitive dysfunction on positive psychotic symptoms - a hypothesis to be examined in future research.

Several limitations warrant consideration. First, data were collected at only two time points across six months, restricting analyses of symptom trajectories and indirect effects involving metacognitive variables. Second, the absence of metric invariance for the MCQ-30 and MSAS, a prerequisite for validly inferring longitudinal relationships (e.g., cross-lagged paths), necessitates cautious interpretation of these longitudinal findings (e.g., inverse association between cognitive self-consciousness at T1 and dissociative symptoms at T2). This caution is further underscored by the possibility that the cross-lagged relationship between positive psychotic and dissociative symptoms may be moderated by unmeasured factors beyond metacognitive dysfunctions, such as trauma exposure. Third, reliance on self-report instruments may introduce bias. Although validity checks were applied, self-reports remain subjective. Future studies should incorporate interviewer‑rated assessments and/or clinician‑administered measures to corroborate diagnostic status and symptom severity, thereby mitigating reporting bias. Finally, the sample comprised solely Hong Kong residents and was predominantly female, well‑educated, and young, which may limit generalisability to other populations.

To conclude, positive psychotic and dissociative symptoms showed mutual influences over time; after controlling for positive symptoms, lower cognitive self‑consciousness and poorer self‑reflectivity predicted increases in dissociative symptoms. Although not without caveats, this is the first study in non‑clinical adults to demonstrate temporal bidirectionality between these symptom domains and to implicate metacognition both as a predictor of, and outcome associated with, dissociative symptom change. Validation of these findings in clinical populations is an important next step.

## Supplementary Information

Below is the link to the electronic supplementary material.

Supplementary Material 1

## Data Availability

Data is available from the corresponding author upon request.
