# Supplementary material for: Prospective investigation of positive psychotic symptoms, dissociative symptoms, and metacognitive dysfunctions in a non-clinical population
Source: Sci Rep. 2025 Oct 13;15:35649. doi: 10.1038/s41598-025-19547-w (PMC12518616; doi:10.1038/s41598-025-19547-w)
Supplement: Supplementary file 1 — Supplementary Material 1 [file 41598_2025_19547_MOESM1_ESM.pdf]

Supplementary information

Measurement invariance across baseline and follow-up

Positive psychotic symptoms

Metric invariance of CAPE-P15 ($\Delta\chi 2 = 2.167$, $\Delta df = 3$, $p = 0.539$) held across time. CFA revealed good model fit for metric invariance of CAPE-P15 ($\chi 2$ [3] = 2.569, $p = 0.463$, CFI = 1.000, RMSEA = 0.000, SRMR = 0.017).

Dissociative symptoms

Metric invariance of DEMO ($\Delta\chi 2 = 10.639$, $\Delta df = 5$, $p = 0.059$) held across time. CFA revealed good model fit for metric invariance of DEMO ($\chi 2$ [15] = 200.801, $p < 0.001$, CFI = 0.974, RMSEA = 0.072, SRMR = 0.037).

Maladaptive metacognitive beliefs

CFA revealed an acceptable fit for configural invariance of MCQ-30 ($\chi 2$ [790] = 7757.961, $p < 0.001$, CFI = 0.866, RMSEA = 0.061, SRMR = 0.080). However, metric invariance of MCQ-30 across time points did not hold ($\Delta\chi 2 = 54.31$, $\Delta df = 25$, $p < 0.001$).

Metacognitive functioning

CFA revealed an acceptable fit for configural invariance of MSAS ($\chi 2$ [258] = 3143.475, $p < 0.001$, CFI = 0.927, RMSEA = 0.069, SRMR = 0.046). However, metric invariance of MSAS across time points did not hold ($\Delta\chi 2 = 57.8$, $\Delta df = 18$, $p < 0.001$).

Scalar invariance was not supported for all scales. Longitudinal SEM models were built on CFA models with the factor loadings of positive psychotic symptoms and dissociative symptoms being invariant across time and the factor loadings of indicators of MCQ-30 and MSAS unconstrained in the respective models.
